# Correction: Structural insights into a bacterial terpene cyclase fused with haloacid dehalogenase-like phosphatase

**DOI:** 10.1039/d5sc90258d

**Published:** 2025-11-26

**Authors:** Keisuke Fujiyama, Hiroshi Takagi, Nhu Ngoc Quynh Vo, Naoko Morita, Toshihiko Nogawa, Shunji Takahashi

**Affiliations:** a Natural Product Biosynthesis Research Unit, RIKEN Center for Sustainable Resource Science Wako Saitama 351-0198 Japan shunjitaka@riken.jp; b Molecular Structure Characterization Unit, RIKEN Center for Sustainable Research Science Wako Saitama 351-0198 Japan

## Abstract

Correction for ‘Structural insights into a bacterial terpene cyclase fused with haloacid dehalogenase-like phosphatase’ by Keisuke Fujiyama *et al.*, *Chem. Sci.*, 2025, **16**, 15310–15319, https://doi.org/10.1039/d5sc04719f.

Upon publication of the original article, the authors were made aware that the preprint by Osika *et al.* (ref. 57 in our original manuscript) had been published,^1^ and is closely related to our study on drimenol synthase from *Aquimarina spongiae* (AsDMS). We hereby correct the sentence on page 15311, left column, line 20, as follows: In this study, we report the co-crystallographic analyses of AsDMS, an enzyme that converts substrate **1** into product **2**, and the biochemical characterization of site-specific variants. During the preparation of this manuscript, Osika *et al.*^1^ disclosed the structure of AsDMS in complex with a non-physiological ligand. In this report, we obtained co-crystal structures of the AsDMS-**1** and AsDMS-**3** complexes. The obtained co-crystal structures of AsDMS represent the first experimentally determined physiological substrate-bound structures of a HAD-TCβ enzyme, revealing distinct substrate-binding pockets for the HAD and TCβ domains.

The co-crystal structures reported in this study involve the physiological substrate, whereas the physiological substrate-free structure of AsDMS was independently reported contemporaneously by Osika *et al.*,^1^ highlighting the complementary nature of these studies. Based on ^18^O-labelling, MESG assays, and the substrate-free crystal structure, Osika *et al.*^1^ proposed a mechanism that releases Pi in a stepwise manner. In the present study, we elucidate the catalytic mechanism (Fig. 6) through co-crystal structures with the physiological substrates and comprehensive site-directed mutagenesis analyses, providing firm experimental evidence for the catalytic process.

The Royal Society of Chemistry apologises for these errors and any consequent inconvenience to authors and readers.
